# Therapeutic drug monitoring of mycophenolic acid and azole antifungals on two distinct LC-MS/MS instruments

**DOI:** 10.1016/j.jmsacl.2024.06.001

**Published:** 2024-06-12

**Authors:** Jill K. Wolken, Wenjing Cao, Min Cui, Zhicheng Jin

**Affiliations:** a University of Wisconsin Hospital and Clinics, Madison, WI, United States of America; b Department of Pathology and Laboratory Medicine, University of Wisconsin – Madison, Madison, WI, United States of America; c Department of Medicine, Case Western Reserve University, United States of America

**Keywords:** Antifungal, Mycophenolate mofetil, Immunosuppressant, Therapeutic drug monitoring, Liquid chromatography, Mass spectrometry

## Abstract

**Background:**

As an active metabolite of a commonly prescribed immunosuppressant, mycophenolic acid (MPA) levels are often monitored to prevent organ rejection following a transplant. Triazoles are often prescribed for treatment of invasive fungal infections in immunocompromised patients. Due to the variability in individual pharmacokinetics and drug-drug interactions, therapeutic drug monitoring is recommended for triazole antifungals. A multiplex LC-MS/MS assay has been developed that can quantify both MPA and triazole drugs in serum.

**Methods:**

A sample preparation procedure was established to spike in internal standard compounds and precipitate proteins. Reversed-phase chromatographic separation was performed on a C18 column with an analysis time of five minutes per sample. The mass spectrometer was operated in multiple reaction monitoring mode. The method was validated on two HPLC systems interfaced with either a Triple Quad 6500 or an API 4000 instrument.

**Results:**

The multiplex assay was linear over a wide dynamic range with analyte measurable ranges of 0.4–48 μg/mL for MPA, 0.1–12 μg/mL for posaconazole, and 0.2–24 μg/mL for voriconazole, itraconazole, hydroxyitraconazole, and isavuconazole. The between-day and intraday imprecisions were less than 10 %. Limits of detection were below 0.04 ug/mL with limits of quantitation below 0.2 μg/mL. Method comparison studies against the current in-house method met acceptance criteria. The instrument comparison study demonstrated a strong correlation between data collected from the two systems.

**Conclusion:**

A robust multiplex LC-MS/MS assay was developed and validated for monitoring MPA and triazoles drug levels in a clinical laboratory. The assay performance on two distinct instruments was acceptable and comparable.

## Highlights

• We developed and validated a fast, sensitive, and robust multiplex mycophenolic acid and antifungal assay.
• Mass spectrometer conditions and assay sensitivities on Triple Quad 6500 and API 4000 instruments were compared and evaluated.
• Considerations and advantages of mass spectrometry-based multiplex assay for therapeutic drug monitoring are discussed.

## Introduction

Therapeutic drug monitoring (TDM) serves as a critical tool to optimize therapy for favorable treatment outcomes [1]. This practice is particularly relevant for drugs that exhibit significant pharmacokinetic variability and operate within a narrow therapeutic window. Notably, immunosuppressants and antifungals are two commonly prescribed classes of drugs that require TDM, especially among immunocompromised patients, such as recipients of solid organ transplants (SOT) [2], [3]. Such monitoring ensures that drug levels remain within the therapeutic range, minimizing the risk of toxicity while maximizing therapeutic efficacy.

Mycophenolate mofetil (MMF) stands as a foundational drug in the maintenance of immunosuppression regimes following solid organ transplantation, chiefly due to its efficacy in preventing acute rejection without inducing renal toxicity [4]. Additionally, enteric-coated mycophenolate sodium is available in a delayed-release formulation designed to minimize gastrointestinal side effects. Upon oral administration, both MMF and mycophenolate sodium are rapidly metabolized to mycophenolic acid (MPA), a selective and noncompetitive inhibitor of inosine-5′-monophosphate dehydrogenase. This inhibition effectively blocks the proliferation of T- and B-cells [5].

The pharmacokinetic variability observed in special populations [6] and the risk of solid organ rejection due to suboptimal MPA blood concentrations [7], [8] highlight the importance of therapeutic monitoring of MPA levels [9]. Notably, MPA does not distribute into red blood cells, making serum or plasma the preferred samples for monitoring, unlike for other immunosuppressants.

Triazoles represent a predominant class of antifungal drugs, often prescribed for the prevention or treatment of invasive fungal infections, such as aspergillosis [10]. Inadequate serum concentrations of these drugs have been implicated in poor patient outcomes and the emergence of drug resistance [10], [11], [12]. Conversely, supratherapeutic serum concentrations can significantly increase the risk of drug-related toxicity [13], [14]. For instance, voriconazole is known to inhibit the activities of CYP2C19, CYP3A4, and CYP2C9, which can lead to numerous clinically relevant drug-drug interactions and toxicities [14]. Given these concerns, TDM is recommended for most triazoles (with the exception of fluconazole) especially in SOT recipients or patients who are otherwise immunocompromised.

Liquid chromatography-tandem mass spectrometry (LC-MS/MS) has gained prominence as a pivotal tool in TDM of both MPA and various antifungals, albeit traditionally these assays have been conducted separately [1], [15], [16]. A recent study extended this capability, demonstrating the simultaneous analysis of MPA alongside three triazoles in a panel specifically designed for lung transplant recipients using LC-MS/MS [17]. Our laboratory previously developed a quantitative assay for MPA using high-performance liquid chromatography (HPLC) equipped with an ultraviolet–visible absorbance detector in 2003. However, due to the obsolescence and lack of manufacturer support for this equipment, it became necessary to transition the MPA assay to another instrument. Additionally, the original antifungal assay on our LC-MS/MS platform required modifications due to its lengthy chromatography separation time of about 10 min, combined with an inadequate number of data points across the chromatographic peak.

Faced with the low test volume for MPA and the aforementioned technical issues, we decided to integrate MPA into the existing antifungal assay panel. To ensure continuous in-house testing capabilities, even when one instrument is out of service, we commonly validate clinical assays on two instruments.

**Scheme 1:**
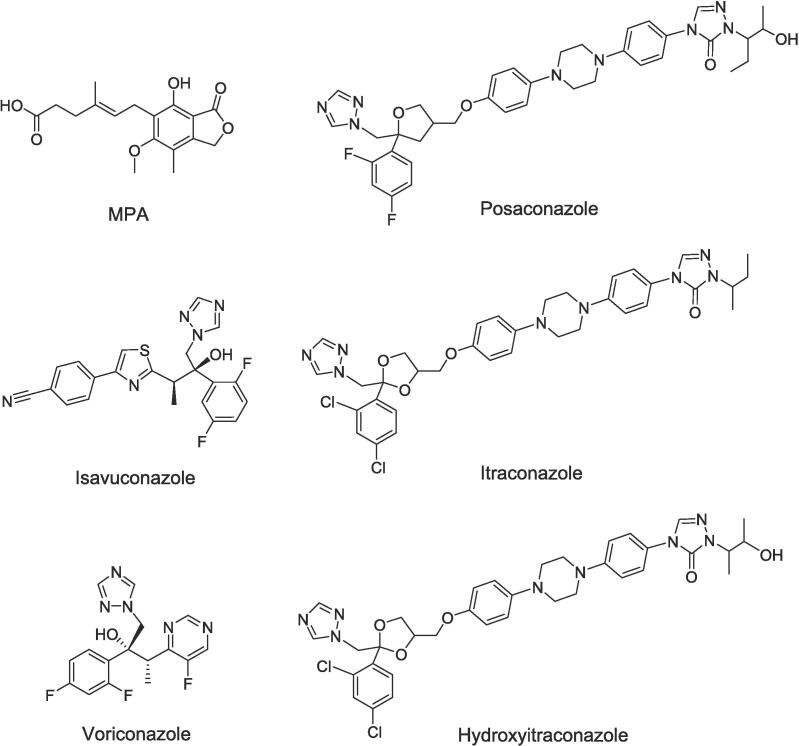
Chemical structures of analytes.

In this context, we are pleased to report the development of a robust LC-MS/MS assay that is capable of simultaneously quantifying MPA, four triazoles (voriconazole, posaconazole, isavuconazole, and itraconazole), and one active metabolite (hydroxyitraconazole) (Scheme 1).

## Materials and Methods

### Reagents

Antifungals (except isavuconazole), mycophenolic acid, and stable isotope labeled internal standards, ^2^H_4_-itraconazole, ^2^H_4_-hydroxyitraconazole, ^2^H_4_-posaconazole, ^2^H_3_-voriconazole, ^2^H_3_-mycophenolic acid, and ^2^H_4_-Isavuconazole were purchased from Cerilliant (Round Rock, TX, USA). Isavuconazole was obtained from Toronto Research Company (North York, ON, Canada). Lyphochek drug-free serum was acquired from Bio-Rad (Hercules, CA, USA). HPLC grade methanol and acetonitrile were purchased from Sigma-Aldrich (St. Louis, MO, USA). Ammonium formate, formic acid, HPLC grade water were purchased from Fisher Scientific (Hampton, NH, USA).

### Sample procurement and preparation

Sample preparation for the LC-MS/MS analysis involved using a solvent containing deuterium-labeled internal standards dissolved in 100 % acetonitrile at a concentration of 200 ng/mL. For each test, 25 µl of either calibrators, controls, or serum samples were combined with 300 µL of the sample preparation solvent. This mixture was then briefly vortexed and subsequently centrifuged at 13,000 g for one minute to facilitate protein precipitation.

Post-centrifugation, 25 µL of the supernatant were carefully transferred to an autosampler vial and further diluted with 300 µL of a dilution solvent composed of 25 % (v/v) acetonitrile in water. The processed samples were stored in the refrigerator before analysis.

This study utilized only fully anonymized patient samples that were not obtained specifically for use in this study through an interaction or intervention with living individuals. Neither informed consent nor IRB review was required.

### Calibrator and quality control preparation

Calibrators and quality controls (QC) were prepared in-house by spiking drug standards into drug-free serum. The calibrator concentrations were 0.2, 0.5, 2, 6, 12, 24 μg/mL for voriconazole, itraconazole, hydroxyitraconazole, and isavuconazole. The calibrator concentrations for posaconazole and mycophenolic acid were 0.1, 0.25, 1, 3, 6, 12 μg/mL, and 0.4, 1, 4, 12, 24, 48 μg/mL, respectively. Drug standards from a different lot were used to prepare QC. The QC of posaconazole was prepared at the concentration of 0.25, 1, and 6 μg/mL. The QC of MPA was 1.5, 4.5, and 25 µg/mL, and the QC of the remaining analytes was at the concentration of 0.5, 2, and 12 μg/mL.

### LC-MS/MS conditions

The LC-MS/MS method for this study was carefully developed and subsequently validated using two sophisticated triple quadrupole instruments. The first setup consisted of an API 4000 (SCIEX, Framingham, MA) coupled with an Agilent 1200 HPLC (Agilent, Santa Clara, CA). The second setup involved a Triple Quad 6500 (TQ 6500) paired with an Agilent 1260 HPLC.

**Fig. 1:**
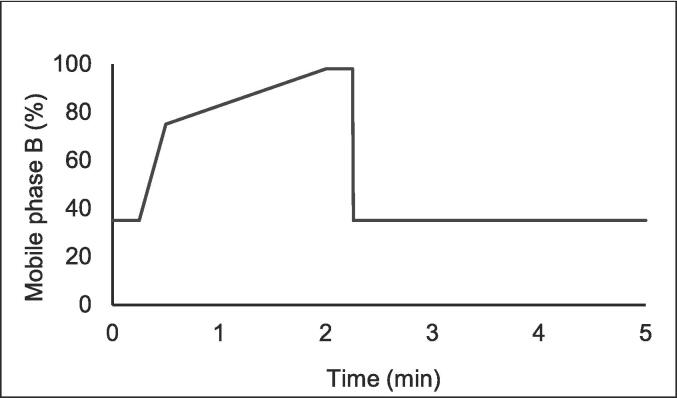
Illustration of HPLC gradient.

Chromatographic separation was achieved using an Ultra C18 analytical column (3 µm, 50 x 2.1 mm) from Restek (Bellefonte, PA), which was outfitted with a corresponding guard column. The mobile phase comprised 10 mM ammonium formate with 0.1 % formic acid (A) and acetonitrile with 0.1 % formic acid (B). A segmented gradient elution was used and is depicted in Fig. 1. Briefly, at a flow rate of 0.4 mL/min, mobile phase B was held at 35 % for 0.25 min, increased to 75 % at 0.5 min, and to 98 % at 2 min. After 0.25 min, the column was equilibrated with 35 % B. Total run time was 5 min for each sample.

The mass spectrometers in this study were equipped with an electrospray ionization (ESI) source, operated in positive ion mode. ESI source parameters were the same for both instruments with source temperature held at 550 °C, spray voltage at 5 kV, curtain gas flow at 35, and collision gas at 8. Two multiple reaction monitoring (MRM) transitions were monitored for each compound. Declustering potential (DP), collision energy (CE), and collision cell exit potential (CXP) were tuned on each instrument as shown in Table 1. Entrance potential was held at 10 V, and cycle time was maintained at 0.5 s. The injection volume was 5 µL on the TQ 6500 and 15 µL on the API 4000. Calibrators and quality controls were extracted with patients’ samples in every batch. Collected data were processed using MultiQuant software (SCIEX, Framingham, MA).

**Table 1:** MRM transitions and parameters.

ID	Precursor	Fragment	TQ 6500			API 4000		
			DP ^c^	CE ^d^	CXP ^e^	DP	CE	CXP
Hydroxyitraconazole 1^a^	721.2	408.2	106	51	10	106	47	12
Hydroxyitraconazole 2^b^	721.2	430.1		51	14		47	6
Hydroxyitraconazole IS	725.2	412.2		51	10		47	12
Isavuconazole 1	438.1	215.0	81	27	18	66	27	14
Isavuconazole 2	438.1	127.0		67	18		73	8
Isavuconazole IS	442.1	219.0		27	18		27	14
Itraconazole 1	705.2	392.2	30	51	10	81	49	18
Itraconazole 2	705.2	432.0		45	6		47	6
Itraconazole IS	709.2	396.2		51	10		49	18
MPA 1	321.0	207.1	56	31	6	56	27	14
MPA 2	321.0	159.0		47	18		49	10
MPA IS1	324.0	210.1		31	6		27	14
MPA IS2	324.0	161.0		47	18		49	10
Posaconazole 1	701.3	683.3	31	45	22	56	45	22
Posaconazole 2	701.3	614.3		47	8		47	10
Posaconazole IS	705.3	687.3		45	22		45	22
Voriconazole 1	350.1	281.0	51	25	16	51	25	20
Voriconazole 2	350.1	127.1		47	16		47	8
Voriconazole IS	353.1	284.0		25	16		25	20

a: ion 1 is the quantifier ion; b: ion 2 is the qualifier ion; c: declustering potential (volts); d: collisional energy (volts); e: collision cell exit potential (volts).

### Method validation

Three quality controls spiked with low, medium, and high concentrations of analytes were used to assess the intraday and between-day imprecision. For intraday imprecision, controls were extracted 20 times within a day and analyzed on both instruments. To assess between-day imprecision, controls were extracted four times per day over 5 days. The precision study was deemed acceptable if the percentage CV was less than 15 %.

To assess accuracy, a method comparison study was conducted against existing in-house tests. The limit of detection (LOD) was calculated as the mean concentration of blank serum plus two standard deviations after running a blank serum five times per day for two days. The lower limit of quantitation (LOQ) was set at a concentration at which the total CV was less than 20 %. The acceptable analyte measurable range (AMR) was defined by analyzing six levels of calibrators in triplicate. AMR was acceptable if the measured concentration of each level was within ± 20 % of the targeted concentration.

Carryover was evaluated by repeated injections of lowest calibrators (L1, L2, L3, L4) immediately after injections of highest calibrators (H1, H2, H3). The carryover was calculated using Equation (1) and < 1 % was considered acceptable [18]. Interferences from hemolyzed, lipemic, and icteric samples were assessed for each analyte. Three sets of specimens were prepared using five patient samples and three quality controls. Each set of eight samples was spiked with either hemoglobin to a final concentration of 1000 mg/dL, intralipid to a final concentration of 1500 mg/dL, or unconjugated bilirubin (icterus) to a final concentration of 40 mg/dL. Results obtained for spiked vs. non-spiked samples were compared. Matrix effect was performed by post-column infusion of internal standard solution and assessing reduction or enhancement in signal intensity for each analyte. Per laboratory policy, when stored in ambient conditions, samples need to be analyzed within 8 h. The sample storage stability was evaluated under two conditions, refrigerator (4 to 9 ˚C) and freezer (−18 to −25 ˚C). Over 200 remnant serum samples and College of American Pathologists (CAP) survey material from 2021 to 2022 stored in freezers for up to four years were reanalyzed to evaluate accuracy of the new assay by comparing against the current in-house method.

(1) $$Carryover\left(\%\right)=\frac{L1-(L3+L4)÷2}{(H2+H3)÷2-(L3+L4)÷2}\times 100\%$$

## Results and discussion

### General consideration

LC-MS/MS with electrospray ionization is increasingly recognized as a robust and powerful tool in the fields of clinical toxicology, endocrinology, and TDM within laboratory medicine. It offers notable advantages such as high specificity, sensitivity, and a broad linear dynamic range. However, LC-MS/MS systems are typically costly, with prices ranging from $200,000 to $500,000 [19].

Given the substantial investment required for these instruments, it is practical to have multiple validated tests available on one LC-MS/MS system to ensure continuity of operations should another instrument experience downtime. The reality for many clinical laboratories, however, is that procuring two identical mass spectrometers may not be feasible due to budget constraints. This necessitates that laboratory scientists develop assays that can operate across instruments from different generations or manufacturers.

When expanding the repertoire of assays on any given instrument, it is crucial to consider compatibility issues related to analytical columns, mobile phases, and the instruments themselves. Owing to these challenges, the development of multiple-analyte assays represents a cost-effective strategy. Furthermore, when the specimen type and sample preparation procedures are compatible, multiplex assays become a viable and efficient option. Here, we have demonstrated the development of a multiplex TDM assay on two distinct instruments, showcasing a practical approach under the constraints typical within clinical laboratory settings.

### LC-MS/MS method development

The API 4000 and TQ 6500, both from the same manufacturer, represent different generations of equipment. All analytes required manual tuning on each instrument to achieve optimal sensitivity. Due to generational differences, the collision energies varied slightly as detailed in Table 1. For each analyte, two abundant fragment ions were identified, with ion 1 serving as the quantifier ion and ion 2 as the qualifier ion. It is important that the peak area ratio of the qualifier ion to the quantifier ion agrees with the theoretical ratio within a margin of ± 20 %.

**Fig. 2:**
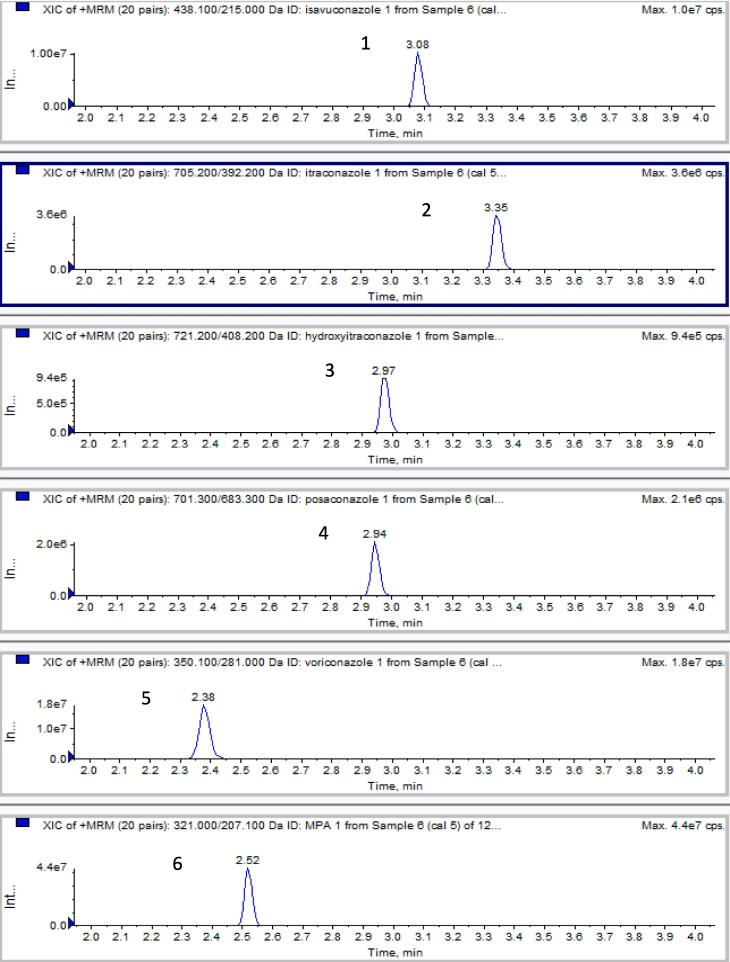
Examples of extracted ion chromatograms of analytes spiked in drug-free serum. Isavuconazole (1), 12 µg/mL; itraconazole (2), 12 µg/mL; hydroxyitraconazole (3), 12 µg/mL; posaconazole (4), 6 µg/mL; voriconazole (5), 12 µg/mL; and MPA (6), 24 µg/mL.

Trained staff closely reviewed the chromatograms to verify correct retention times and ensure accurate identification of analytes. Our first-generation antifungal assay used a Kinetex 2.6 µm C18, 3x100 mm analytical column. To decrease the chromatography separation time for newer assays, we opted for a shorter and narrower C18 column (2.1x50 mm). An example of extracted ion chromatograms, showcasing analytes spiked into drug-free serum (equivalent to a level 5 calibrator), obtained using the Triple Quad 6500, can be seen in Fig. 2.

### Validation data

#### Imprecision and accuracy

As shown in Table 2, the between-day imprecision for both instruments were less than 10 %, while the intraday imprecisions were less than 7 % (data not shown). Both intraday and between-day imprecision levels met the established acceptance criteria.

**Table 2:** Between-day imprecision.

Analyte	Instrument	Low		Med		High	
		Average	CV (%)	Average	CV (%)	Average	CV (%)
Posaconazole	TQ 6500	0.17	8.2	0.88	6.7	5.6	5.1
	API 4000	0.19	7.3	0.95	7.7	5.8	6.8
Voriconazole	TQ 6500	0.45	4.8	1.99	2.2	11.0	2.6
	API 4000	0.45	5.7	2.06	5.4	11.5	4.7
Itraconazole	TQ 6500	0.37	7.3	2.04	4.3	11.2	5.7
	API 4000	0.38	6.4	2.12	6.4	11.6	5.2
Hydroxyitraconazole	TQ 6500	0.44	9.3	1.92	4.9	11.5	5.0
	API 4000	0.42	6.5	2.00	5.3	11.9	5.1
Isavuconazole	TQ 6500	0.37	4.2	1.96	2.7	11.7	2.4
	API 4000	0.37	4.1	2.10	4.2	12.2	5.4
MPA	TQ 6500	1.46	3.3	4.44	3.1	23.6	2.9
	API 4000	1.50	4.9	4.50	5.7	24.2	5.4

The accuracy of the assays was assessed through a comparison involving results from 40 patient specimens. These were analyzed using both the newly validated method and the current in-house method. The correlation coefficients obtained ranged from 0.983 to 0.996. Additionally, Deming regression analysis showed that the slopes across the two instruments varied from 0.87 to 1.1, as outlined in Table 1 in the online Supplemental Data.

Furthermore, Bland-Altman plots were utilized to evaluate the bias for all analytes, revealing that all were within the acceptable range, as shown in Fig. 1, in the online Supplemental Data.

Collectively, these statistical evaluations demonstrated that the newly validated method correlates acceptably with the existing in-house method on both analytical instruments.

#### Sensitivity, analyte measurable range, and linearity

The sensitivity of the test was reflected through the parameters of LOD and limit of quantitation (LOQ). Our analysis indicated that the LOD values were consistently comparable between the two instruments, API 4000 and TQ 6500. However, the LOQ performance was slightly superior on the TQ 6500 compared to the API 4000, as detailed in Table 2 in the online Supplemental Data.

The AMR was determined for various compounds, with posaconazole showing a range of 0.1 to 12 µg/mL. MPA exhibited a broader range from 0.4 to 48 µg/mL. The AMR for other analytes was between 0.2 to 24 µg/mL, as demonstrated in Fig. 2 in the online Supplemental Data.

These outcomes affirm that the method exhibits linearity across a broad dynamic range for all tested analytes on both instruments.

#### Interference, matrix effect, and storage stability

**Fig. 3:**
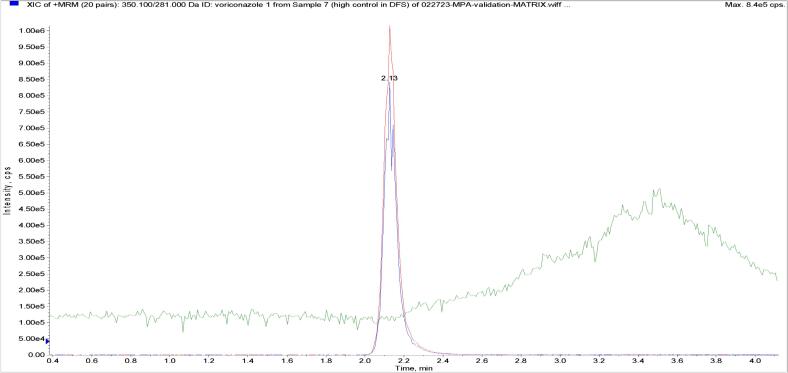
Matrix effect study of voriconazole on the API 4000 instrument using high QC sample.

Interference studies focusing on endogenous sources showed that hemoglobin (1000 mg/dL), bilirubin (40 mg/dL), and lipids (1500 mg/dL) did not affect the quantitation results (Table 3 to Table 5 in the online Supplemental Data). As an illustrative example, the matrix effect study for voriconazole is depicted in Fig. 3. Notably, the internal standard's ion intensity remained unaffected when endogenous analyte eluted from the analytical column, suggesting that the assay was free from any matrix effects under the tested conditions. Further examination revealed that no signal intensity reduction or enhancement occurred for any analytes, which is visually supported in Fig. 3 in the online Supplemental Data.

**Table 3:** Deming regression analysis of results from two instruments (API 4000 ∼ TQ 6500).

	Voriconazole	Posaconazole	Itraconazole	Hydroxy-itraconazole	Isavuconazole	MPA
Slope C.I. ^a^	1.005 0.985–1.025	1.037 1.012–1.059	1.010 0.992–1.023	1.041 0.944–1.155	1.039 0.953–1.124	1.011 0.982–1.047
Intercept C.I.	0.034 −0.016–0.093	−0.007 −0.047–0.0.037	0.023 −0.019–0.069	0.008 −0.177–0.149	−0.014 −0.213–0.169	−0.007 −0.084–0.069
R	0.9979	0.9966	0.9989	0.9815	0.9925	0.9945

Additionally, a carryover study confirmed that any potential carryover was within the acceptable range for all analytes. Sample storage stability was another key aspect assessed during the study. Analyte stability was maintained for up to 15 days in the refrigerator and up to 30 days in the freezer (Fig. 4 in the online Supplemental Data).

#### Instrument comparison

The instrument comparison study showed that correlation coefficients were in the range of 0.9815 to 0.9989 and the slopes were between 1.005 and 1.041 (Table 3). These data demonstrate a strong correlation between results obtained on API 4000 and TQ 6500 systems.

Our laboratory purchased the College of American Pathologists (CAP) antifungal drug monitoring (AFT) and MPA survey samples to assess the performance of our in-house assays for posaconazole, itraconazole, voriconazole, and MPA. Additionally, UK National External Quality Assessment Service (UK NEQAS) antifungal survey samples were procured as proficiency test specimens, specifically for hydroxy-itraconazole.

To verify the accuracy of our multiplex assay across different instruments, we tested the remnant AFT, MPA, and UK NEQAS survey samples that had been stored at −20 ˚C. These samples had been preserved in the freezer for a duration of one to two years. The analytical results from both instruments fell within three standard deviations (SD) of the peer group mean (Table 6 to Table 10 in the online Supplemental Data).

In summary, our laboratory successfully expanded an antifungal panel to include MPA. Initially, the low test volume for MPA made it impractical to develop this assay solely on a mass spectrometry platform, and outsourcing to a reference laboratory would have led to delays in patient care. By developing a multiplex assay and implementing it on our LC-MS/MS system, we were able to keep the testing in-house, thus meeting our target turnaround times.

Additionally, the new assay configuration halved the analysis time from 10 min to 5 min per sample. This reduction in instrument run-time not only increased throughput but also allowed us to add two additional tests to the same instrument, thereby enhancing equipment utilization and reducing the cost per test.

The switch from an ultraviolet–visible flow cell detector to mass spectrometer significantly increased sensitivity. The specimen volume required for the MPA assay was reduced dramatically from 500 µL to just 25 µL. We also optimized the analyte measurement ranges to accommodate the TDM requirements for six analytes without the need for manual sample dilution.

The robust performance of this multiplex assay on both the API 4000 and TQ 6500 instruments underscores the versatility and reliability of the method. The success of this method development and validation serves as a practical model for other clinical laboratories aiming to develop multiplex clinical assays, enhancing their capabilities while optimizing resource usage.

## Conclusion

Our laboratory successfully developed and validated a rapid, sensitive, and robust LC-MS/MS method designed to quantify MPA, voriconazole, posaconazole, isavuconazole, itraconazole, and hydroxyitraconazole for TDM. This multiplex assay, validated on two distinct instruments—the API 4000 and the TQ 6500—demonstrated improved efficiency through shortened chromatographic separation times, which in turn increased throughput, maximized instrument utilization, and significantly reduced the cost per test.

The new assay's wide dynamic range facilitated a straightforward sample preparation procedure, eliminating the need for complex manual dilution steps. This cost-effective multiplex assay has enabled sustainable in-house TDM, proving particularly advantageous for drugs with lower testing volumes.

## CRediT authorship contribution statement

**Jill K. Wolken:** Writing – original draft, Validation, Methodology, Formal analysis, Data curation, Conceptualization. **Wenjing Cao:** Writing – review & editing, Writing – original draft, Visualization, Formal analysis, Data curation. **Min Cui:** Writing – review & editing, Writing – original draft, Visualization, Conceptualization. **Zhicheng Jin:** Writing – review & editing, Writing – original draft, Visualization, Methodology, Formal analysis, Conceptualization.
