# Supplementary material for: Therapeutic drug monitoring of mycophenolic acid and azole antifungals on two distinct LC-MS/MS instruments
Source: J Mass Spectrom Adv Clin Lab. 2024 Jun 12;33:7–13. doi: 10.1016/j.jmsacl.2024.06.001 (PMC11225655; doi:10.1016/j.jmsacl.2024.06.001)
Supplement: Supplementary Data 1 [file mmc1.docx]

**Supplemental Data File**

Therapeutic Drug Monitoring of Mycophenolic Acid and Azole Antifungals on Two Distinct LC-MS/MS Instruments

Jill Wolken ^1^, Wenjing Cao ^1,2^, Min Cui ^3^, Zhicheng Jin ^1,2*^

1. University of Wisconsin Hospital and Clinics, Madison, WI

2. Department of Pathology and Laboratory Medicine, University of Wisconsin – Madison

Madison, WI, USA

3. Department of Medicine, Case Western Reserve University

* Corresponding Author

Present Address:

Zhicheng Jin, PhD

Department of Pathology and Laboratory Medicine

University Wisconsin – Madison

600 Highland Avenue, Madison, WI 53792, United States.

Fax: +1-608-263-1568

Email: zjin237@wisc.edu

**Keywords**

Antifungal, mycophenolate mofetil, immunosuppressant, therapeutic drug monitoring, liquid chromatography, mass spectrometry

**Method Validation Results**

1. Method comparison results

A method comparison study was performed to evaluate accuracy of the validation method. As shown in Supplemental Table 1, slopes and intercepts were determined by curve fitting with Deming regression using the R Program. Bland-Altman plots comparing results from validation method vs. current in-house method were shown in Supplemental Figure 1. These results demonstrated an acceptable correlation between validation method and current method on both instruments.

**Supplemental Table 1**. Deming regression analysis of results (validation method ~ current method)

|  | | Voriconazole | Posaconazole | Itraconazole | Hydroxy-itraconazole | Isavuconazole | MPA |
| --- | --- | --- | --- | --- | --- | --- | --- |
| Sample size | | 40 | 42 | 40 | 40 | 42 | 39 |
| TQ 6500 | Slope  C.I. ^a^ | 1.022  0.943-1.102 | 0.872  0.817-0.950 | 0.963  0.926-0.995 | 1.033  0.995-1.093 | 0.955  0.905-1.036 | 0.975  0.930-1.035 |
|  | Intercept  C.I. | 0.013  -0.131-0.158 | 0.020  -0.094-0.103 | -0.006  -0.047-0.043 | 0.054  -0.032-0.130 | 0.030  -0.130-0.149 | -0.098  -0.302-0.071 |
|  | R | 0.9901 | 0.9882 | 0.9956 | 0.9941 | 0.9875 | 0.9832 |
| API 4000 | Slope  C.I. | 1.078  1.032-1.122 | 0.897  0.836-0.976 | 0.931  0.894-0.956 | 1.094  1.044-1.143 | 0.942  0.886-1.014 | 0.976  0.920-1.053 |
|  | Intercept  C.I. | 0.001  -0.092-0.090 | 0.016  -0.086-0.109 | 0.014  -0.038-0.069 | -0.004  -0.092-0.074 | 0.032  -0.107-0.152 | -0.094  -0.268-0.049 |
|  | R | 0.9942 | 0.985 | 0.996 | 0.995 | 0.988 | 0.9838 |

a: Confidence interval (C.I.)

**B**

**A**

API 4000
Voriconazole

TQ 6500
Voriconazole

API 4000
Voriconazole

TQ 6500
Voriconazole

**D**

**C**

TQ 6500
Posaconazole

API 4000
Posaconazole

**F**

**E**

TQ 6500
Itraconazole

API 4000
Itraconazole

**H**

**G**

TQ 6500
Hydroxyitraconazole

API 4000
Hydroxyitraconazole

**J**

**I**

TQ 6500
Isavuconazole

API 4000
Isavuconazole

**K**

**L**

API 6500

MPA

API 4000
MPA

**Supplemental Figure 1**. Bland-Altman plots comparing validation method vs. reference method.

A: voriconazole, API 4000; B: voriconazole, TQ 6500; C: posaconazole, API 4000; D: posaconazole, TQ 6500; E: itraconazole, API 4000; F: itraconazole, TQ 6500; G: hydroxyitraconazole; API 4000; H: hydroxyitraconazole, TQ 6500; I: isavuconazole, API 4000; J: isavuconazole, TQ 6500; K: MPA, API 4000; L: MPA, TQ 6500.

1. LOD and LOQ

**Supplemental Table 2**. Assay sensitivity of the two systems

| Analyte | Instrument | LOD (µg/mL) | LOQ (µg/mL) |
| --- | --- | --- | --- |
| Posaconazole | TQ 6500 | 0.03 | 0.05 |
|  | API 4000 | 0.04 | 0.04 |
| Voriconazole | TQ 6500 | 0.01 | 0.05 |
|  | API 4000 | 0.02 | 0.1 |
| Itraconazole | TQ 6500 | 0.02 | 0.1 |
|  | API 4000 | 0.01 | 0.1 |
| Hydroxyitraconazole | TQ 6500 | 0.01 | 0.1 |
|  | API 4000 | 0.01 | 0.2 |
| Isavuconazole | TQ 6500 | 0.03 | 0.05 |
|  | API 4000 | 0.01 | 0.1 |
| MPA | TQ 6500 | 0.01 | 0.05 |
|  | API 4000 | 0.02 | 0.04 |

1. AMR of the assay on TQ 6500 and API 4000 instruments

**Supplemental Figure 2**. Analyte measurable ranges of the two instruments

1. Endogenous interference studies

Interferences from hemolyzed, lipemic, and icteric samples were assessed for each analyte. Five patient samples and three quality controls were spiked with hemoglobin to the final concentration of 1000 mg/dL, spiked with intralipid to the concentration of 1500 mg/dL, or spiked with unconjugated bilirubin (icterus) to the final concentration of 40 mg/dL, respectively. Results obtained for spiked vs. non-spiked samples were assessed. Coefficient of correlation and slopes indicated that hemolysis, icterus, and intralipid didn’t affect quantification of all analytes (Table 3 to Table 5 in Supplemental Data).

Supplemental Table 3: Summary of hemolysis interference study

| Analyte | Voriconazole | Posaconazole | Itraconazole | Hydroxyitraconazole | Isavuconazole | MPA |
| --- | --- | --- | --- | --- | --- | --- |
| Slope | 1.0693 | 0.9523 | 0.9371 | 0.9460 | 0.9544 | 0.9626 |
| Intercept | -0.2673 | 0.0341 | -0.0647 | -0.0627 | 0.0332 | -0.0482 |
| R | 0.9989 | 0.9965 | 0.9988 | 0.9996 | 0.9999 | 0.9999 |

Supplemental Table 4: Summary of icterus interference study

| Analyte | Voriconazole | Posaconazole | Itraconazole | Hydroxyitraconazole | Isavuconazole | MPA |
| --- | --- | --- | --- | --- | --- | --- |
| Slope | 0.9768 | 0.9643 | 1.0219 | 0.9657 | 0.9867 | 0.9754 |
| Intercept | 0.0686 | 0.0317 | -0.1148 | 0.0195 | 0.0209 | 0.0352 |
| R | 0.9997 | 0.9992 | 0.9990 | 0.9991 | 0.9996 | 0.9999 |

Supplemental Table 5: Summary of intralipid interference study

| Analyte | Voriconazole | Posaconazole | Itraconazole | Hydroxyitraconazole | Isavuconazole | MPA |
| --- | --- | --- | --- | --- | --- | --- |
| Slope | 0.9982 | 1.0547 | 0.9975 | 0.9695 | 1.0012 | 1.0244 |
| Intercept | 0.0203 | 0.036 | 0.1426 | 0.1181 | -0.0067 | 0.0287 |
| R | 0.9989 | 0.9966 | 0.997 | 0.9992 | 0.9996 | 0.9998 |

1. Matrix effect study

Post column infusion of internal standard solution experiment was performed to assess matrix effect or ion suppression. Five patients’ and one quality control specimen without internal standards were injected via autosampler and separated on analytical column, while the solution containing all six internal standards were continuously infused through the syringe pump. The two liquid flows merged at a T-union and went into mass spectrometer. The goal was to have a minimum drop in internal standard signal at the retention times that correspond to each analyte. This data was obtained on the API 4000 instrument. Our data showed that no signal intensity reduction or enhancement was observed for all analytes (Figure 3 in the online Supplemental Data).

B

A

Posaconazole, IS

Posaconazole, ions 1 and 2

Voriconazole, IS

Voriconazole, ions 1 and 2

C

Isavuconazole, IS

Isavuconazole, ions 1 and 2

D

Itraconazole, IS

Itraconazole, ions 1 and 2

E

Hydroxyitraconazole, IS

Hydroxyitraconazole, ions 1 and 2

F

MPA, IS

MPA,
ions 1 and 2

**Supplemental Figure 3**. Matrix effect study on API 4000 instrument using high QC sample.

A, voriconazole; B, posaconazole; C, isavuconazole; D, itraconazole; E, hydroxyitraconazole; F, MPA.

1. Sample storage stability.

One sample was aliquoted and stored in the refrigerator (4 to 9 ̊C) or the freezer (-18 to -25 ̊C) for 0, 3, 7, and 15 days. Aliquots were analyzed on TQ 6500, and sample storage stability was evaluated. Data showed that no sample degradation was observed after 15 days storage in the refrigerator or 30 days storage in the freezer.

Supplemental Figure 4. Specimen stability study following storage in refrigerator (A) or in freezer (B)

1. Results from remnant survey samples

Supplemental Table 6. Voriconazole results on the two instruments.

| No. | Survey | Sample | survey result | TQ 6500 Result | TQ 6500  s.d. index | API 4000 Result | API 4000  s.d. index |
| --- | --- | --- | --- | --- | --- | --- | --- |
| 1 | NEQAS, 2021 June 20 | 226A VORIC | 1.63 ± 0.16 | 1.75 | 0.75 | 1.66 | 0.00 |
| 2 | NEQAS, 2021 June 20 | 226B VORIC | 4.19 ± 0.34 | 4.38 | 0.56 | 4.30 | 0.32 |
| 3 | NEQAS, 2021 June 20 | 226C VORIC | 5.80 ± 0.68 | 6.04 | 0.35 | 6.18 | 0.56 |
| 4 | CAP, AFD, 2021-B | 21-AFD-04 | 2.14 ± 0.18 | 2.11 | 0.17 | 2.16 | 0.11 |
| 5 | CAP, AFD, 2021-B | 21-AFD-05 | 14.65 ± 1.64 | 15.45 | 0.49 | 15.46 | 0.49 |
| 6 | CAP, AFD, 2021-B | 21-AFD-06 | 6.33 ± 0.57 | 6.38 | 0.09 | 6.31 | 0.04 |
| 7 | NEQAS, 2021 Aug 15 | 227A VORIC | 3.93 ± 0.35 | 3.87 | 0.17 | 3.76 | 0.49 |
| 8 | NEQAS, 2021 Aug 15 | 227B VORIC | 2.06 ± 0.20 | 2.10 | 0.2 | 1.98 | 0.40 |
| 9 | NEQAS, 2021 Aug 15 | 227C VORIC | 5.89 ± 0.41 | 6.11 | 0.54 | 6.20 | 0.76 |
| 10 | NEQAS, 2021 Oct 17 | 228A VORIC | 0 | 0.00 | 0 | 0.00 | 0.00 |
| 11 | NEQAS, 2021 Oct 17 | 228B VORIC | 8.48 ± 0.68 | 9.75 | 1.87 | 9.35 | 1.28 |
| 12 | NEQAS, 2021 Oct 17 | 228C VORIC | 0.78 ± 0.08 | 0.84 | 0.75 | 0.78 | 0.00 |
| 13 | NEQAS, 2022 Feb 02 | 229A VORIC | 4.00 ± 0.37 | 4.47 | 1.27 | 4.55 | 1.49 |
| 14 | NEQAS, 2022 Feb 02 | 229B VORIC | 1.11 ± 0.10 | 1.16 | 0.5 | 1.18 | 0.70 |
| 15 | NEQAS, 2022 Feb 02 | 229C VORIC | 7.21 ± 0.45 | 7.76 | 1.22 | 7.89 | 1.51 |
| 16 | NEQAS, 2022 Feb 07 | 230A VORIC | 4.00 ± 0.33 | 4.38 | 1.15 | 4.10 | 0.30 |
| 17 | NEQAS, 2022 Feb 07 | 230B VORIC | 2.11 ± 0.20 | 2.36 | 1.25 | 2.31 | 1.00 |
| 18 | NEQAS, 2022 Feb 07 | 230C VORIC | 6.03 ± 0.37 | 6.64 | 1.65 | 6.53 | 1.35 |
| 19 | NEQAS, 2022 April 11 | 231A VORIC | 0 | 0.00 | 0 | 0.00 | 0.00 |
| 20 | NEQAS, 2022 April 11 | 231B VORIC | 3.09 ± 0.33 | 3.59 | 1.52 | 3.78 | 2.09 |
| 21 | NEQAS, 2022 April 11 | 231C VORIC | 5.20 ± 0.44 | 5.80 | 1.36 | 5.54 | 0.77 |
| 22 | CAP, AFD, 2022-B | 22-AFD-04 | 1.09 ± 0.11 | 1.22 | 1.18 | 1.23 | 1.27 |
| 23 | CAP, AFD, 2022-B | 22-AFD-05 | 15.98 ± 1.54 | 18.10 | 1.38 | 18.39 | 1.56 |
| 24 | CAP, AFD, 2022-B | 22-AFD-06 | 5.29 ± 0.44 | 6.25 | 2.18 | 5.90 | 1.39 |

Supplemental Table 7. Posaconazole results on the two instruments.

| No. | Survey | Sample | Survey result | TQ 6500 Result | TQ 6500  s.d. index | API 4000 Result | API 4000  s.d. index |
| --- | --- | --- | --- | --- | --- | --- | --- |
| 1 | NEQAS, 2021 June 20 | 226 A POSA | 4.54 ± 0.38 | 4.54 | 0.00 | 4.72 | 0.47 |
| 2 | NEQAS, 2021 June 20 | 226 B POSA | 6.02 ± 0.57 | 6.17 | 0.26 | 6.07 | 0.09 |
| 3 | NEQAS, 2021 June 20 | 226C POSA | 0.98 ± 0.20 | 1.01 | 0.03 | 1.07 | 0.45 |
| 4 | NEQAS, 2021 Aug 15 | 227A POSA | 2.21 ± 0.20 | 2.11 | 0.50 | 2.32 | 0.55 |
| 5 | NEQAS, 2021 Aug 15 | 227B POSA | 3.02 ± 0.23 | 2.95 | 0.30 | 3.08 | 0.26 |
| 6 | NEQAS, 2021 Aug 15 | 227C POSA | 0.52 ± 0.07 | 0.54 | 0.29 | 0.56 | 0.57 |
| 7 | NEQAS, 2021 Oct 17 | 228A POSA | 1.34 ± 0.15 | 1.33 | 0.07 | 1.37 | 0.20 |
| 8 | NEQAS, 2021 Oct 17 | 228B POSA | 4.85 ± 0.49 | 4.92 | 0.14 | 4.98 | 0.27 |
| 9 | NEQAS, 2021 Oct 17 | 228C POSA | 0.46 ± 0.07 | 0.47 | 0.14 | 0.47 | 0.14 |
| 10 | NEQAS, 2022 Feb 02 | 229A POSA | 1.19 ± 0.13 | 1.20 | 0.08 | 1.29 | 0.77 |
| 11 | NEQAS, 2022 Feb 02 | 229B POSA | 4.44 ± 0.36 | 4.66 | 0.61 | 4.91 | 1.31 |
| 12 | NEQAS, 2022 Feb 02 | 229C POSA | 0.79 ± 0.08 | 0.83 | 0.50 | 0.84 | 0.63 |
| 13 | NEQAS, 2022 Feb 07 | 230A POSA | 0.30 ± 0.04 | 0.29 | 0.25 | 0.30 | 0.00 |
| 14 | NEQAS, 2022 Feb 07 | 230B POSA | 3.07 ± 0.21 | 3.19 | 0.57 | 3.40 | 1.57 |
| 15 | NEQAS, 2022 Feb 07 | 230C POSA | 4.36 ± 0.31 | 4.79 | 1.39 | 5.03 | 2.16 |
| 16 | NEQAS, 2022 April 11 | 231A POSA | 2.28 ± 0.18 | 2.27 | 0.06 | 2.64 | 2.00 |
| 17 | NEQAS, 2022 April 11 | 231B POSA | 5.24 ± 0.55 | 5.64 | 0.73 | 5.76 | 0.95 |
| 18 | NEQAS, 2022 April 11 | 231C POSA | 0 | 0 | 0.00 | 0.00 | 0.00 |
| 19 | CAP, AFD, 2021-B | 21-AFD-04 | 1.09 ± 0.12 | 1.11 | 0.17 | 1.15 | 0.50 |
| 20 | CAP, AFD, 2021-B | 21-AFD-05 | 4.11 ± 0.40 | 4.2 | 0.23 | 4.32 | 0.53 |
| 21 | CAP, AFD, 2021-B | 21-AFD-06 | 7.09 ± 1.01 | 7.76 | 0.66 | 8.31 | 1.21 |
| 22 | CAP, AFD, 2022-B | 22-AFD-04 | 0.94 ± 0.12 | 1.07 | 1.08 | 1.12 | 1.50 |
| 23 | CAP, AFD, 2022-B | 22-AFD-05 | 0.48 ± 0.07 | 0.54 | 0.86 | 0.54 | 0.86 |
| 24 | CAP, AFD, 2022-B | 22-AFD-06 | 2.45 ± 0.29 | 2.72 | 0.93 | 2.72 | 0.93 |

Supplemental Table 8. Itraconazole results on the two instruments.

| No. | Survey | Sample | survey result | TQ 6500 Result | TQ 6500  s.d. index | API 4000 Result | API 4000  s.d. index |
| --- | --- | --- | --- | --- | --- | --- | --- |
| 1 | NEQAS, 2021 June 20 | 226A ITRA | 5.44 ± 0.58 | 6.09 | 1.12 | 5.99 | 0.95 |
| 2 | NEQAS, 2021 June 20 | 226B ITRA | 3.06 ± 0.28 | 3.22 | 0.57 | 3.38 | 1.14 |
| 3 | NEQAS, 2021 June 20 | 226C ITRA | 1.64 ± 0.14 | 1.76 | 0.86 | 1.75 | 0.79 |
| 4 | CAP, AFD, 2021-B | 21-AFD-04 | 4.62 ± 0.82 | 4.39 | 0.28 | 4.35 | 0.33 |
| 5 | CAP, AFD, 2021-B | 21-AFD-05 | 8.98 ± 1.76 | 8.21 | 0.44 | 8.30 | 0.39 |
| 6 | CAP, AFD, 2021-B | 21-AFD-06 | 9.36 ± 2.89 | 11.09 | 0.60 | 11.28 | 0.66 |
| 7 | NEQAS, 2021 Aug 15 | 227A ITRA | 0.76 ± 0.06 | 0.81 | 0.83 | 0.75 | 0.17 |
| 8 | NEQAS, 2021 Aug 15 | 227B ITRA | 4.47 ± 0.34 | 4.71 | 0.71 | 4.81 | 1.00 |
| 9 | NEQAS, 2021 Aug 15 | 227C ITRA | 1.36 ± 0.11 | 1.43 | 0.64 | 1.49 | 1.18 |
| 10 | NEQAS, 2021 Oct 17 | 228A ITRA | 1.39 ± 0.14 | 1.54 | 1.07 | 1.54 | 1.07 |
| 11 | NEQAS, 2021 Oct 17 | 228B ITRA | 0.70 ± 0.07 | 0.74 | 0.57 | 0.75 | 0.71 |
| 12 | NEQAS, 2021 Oct 17 | 228C ITRA | 0.37 ± 0.04 | 0.35 | 0.50 | 0.39 | 0.50 |
| 13 | NEQAS, 2022 Feb 02 | 229A ITRA | 2.75 ± 0.23 | 3.22 | 2.04 | 3.05 | 1.30 |
| 14 | NEQAS, 2022 Feb 02 | 229B ITRA | 0.68 ± 0.06 | 0.76 | 1.33 | 0.70 | 0.33 |
| 15 | NEQAS, 2022 Feb 02 | 229C ITRA | 4.85 ± 0.50 | 5.65 | 1.60 | 5.66 | 1.62 |
| 16 | NEQAS, 2022 Feb 07 | 230A ITRA | 0.44 ± 0.04 | 0.44 | 0.00 | 0.48 | 1.00 |
| 17 | NEQAS, 2022 Feb 07 | 230B ITRA | 2.05 ± 0.15 | 2.34 | 1.93 | 2.21 | 1.07 |
| 18 | NEQAS, 2022 Feb 07 | 230C ITRA | 4.10 ± 0.45 | 4.5 | 0.89 | 4.65 | 1.22 |
| 19 | NEQAS, 2022 April 11 | 231A ITRA | 0.66 ± 0.05 | 0.68 | 0.40 | 0.72 | 1.20 |
| 20 | NEQAS, 2022 April 11 | 231B ITRA | 2.41 ± 0.25 | 2.55 | 0.56 | 2.69 | 1.12 |
| 21 | NEQAS, 2022 April 11 | 231C ITRA | 1.09 ± 0.07 | 1.19 | 1.43 | 1.24 | 2.14 |
| 22 | CAP, AFD, 2022-B | 22-AFD-04 | 0.71 ± 0.11 | 0.74 | 0.27 | 0.74 | 0.27 |
| 23 | CAP, AFD, 2022-B | 22-AFD-05 | 1.38 ± 0.21 | 1.48 | 0.48 | 1.47 | 0.43 |
| 24 | CAP, AFD, 2022-B | 22-AFD-06 | 5.62 ± 0.78 | 6.56 | 1.21 | 6.84 | 1.56 |

Supplemental Table 9. Hydroxyitraconazole results on the two instruments.

| No. | Survey | Sample | survey result | TQ 6500 Result | TQ 6500  s.d. index | API 4000 Result | API 4000  s.d. index |
| --- | --- | --- | --- | --- | --- | --- | --- |
| 1 | NEQAS, 2021 June 20 | 226A ITRA | 3.80 ± 0.44 | 3.89 | 0.02 | 3.88 | 0.18 |
| 2 | NEQAS, 2021 June 20 | 226A ITRA | 2.83 ± 0.35 | 2.70 | 0.37 | 2.75 | 0.23 |
| 3 | NEQAS, 2021 June 20 | 226C ITRA | 1.14 ± 0.08 | 1.11 | 0.38 | 1.05 | 1.13 |
| 4 | NEQAS, 2021 Aug 15 | 227A ITRA | 0.71 ± 0.07 | 0.70 | 0.14 | 0.75 | 0.57 |
| 5 | NEQAS, 2021 Aug 15 | 227B ITRA | 2.10 ± 0.20 | 2.03 | 0.35 | 2.14 | 0.20 |
| 6 | NEQAS, 2021 Aug 15 | 227C ITRA | 3.12 ± 0.28 | 3.23 | 0.39 | 3.37 | 0.89 |
| 7 | NEQAS, 2021 Oct 17 | 228A ITRA | 1.37 ± 0.15 | 1.50 | 0.87 | 1.31 | 0.40 |
| 8 | NEQAS, 2021 Oct 17 | 228B ITRA | 2.02 ± 0.34 | 1.98 | 0.12 | 2.09 | 0.21 |
| 9 | NEQAS, 2021 Oct 17 | 228C ITRA | 0.76 ± 0.07 | 0.75 | 0.14 | 0.76 | 0.00 |
| 10 | NEQAS, 2022 Feb 02 | 229A ITRA | 2.65 ± 0.36 | 2.54 | 0.31 | 2.77 | 0.33 |
| 11 | NEQAS, 2022 Feb 02 | 229B ITRA | 1.38 ± 0.18 | 1.39 | 0.06 | 1.43 | 0.28 |
| 12 | NEQAS, 2022 Feb 02 | 229C ITRA | 2.00 ± 0.24 | 2.07 | 0.29 | 2.09 | 0.38 |
| 13 | NEQAS, 2022 Feb 07 | 230A ITRA | 0.42 ± 0.03 | 0.40 | 0.67 | 0.34 | 2.67 |
| 14 | NEQAS, 2022 Feb 07 | 230B ITRA | 1.66 ± 0.14 | 1.68 | 0.14 | 1.65 | 0.07 |
| 15 | NEQAS, 2022 Feb 07 | 230C ITRA | 2.07 ± 0.18 | 1.94 | 0.72 | 2.27 | 1.11 |
| 16 | NEQAS, 2022 April 11 | 231A ITRA | 0.72 ± 0.04 | 0.70 | 0.5 | 0.70 | 0.50 |
| 17 | NEQAS, 2022 April 11 | 231B ITRA | 1.79 ± 0.15 | 1.73 | 0.4 | 1.82 | 0.20 |
| 18 | NEQAS, 2022 April 11 | 231C ITRA | 3.16 ± 0.23 | 3.24 | 0.35 | 3.55 | 1.70 |

Supplemental Table 10. MPA results on the two instruments.

| No. | Survey | Sample | survey result | TQ 6500 Result | TQ 6500  s.d. index | API 4000 Result | API 4000  s.d. index |
| --- | --- | --- | --- | --- | --- | --- | --- |
| 1 | CAP, MPA, 2021-B | 21 - 4 MPA | 4.32 ± 0.3 | 4.65 | 1.10 | 4.53 | 0.70 |
| 2 | CAP, MPA, 2021-B | 21 - 5 MPA | 3.42 ± 0.27 | 3.63 | 0.78 | 3.85 | 1.59 |
| 3 | CAP, MPA, 2021-B | 21 - 6 MPA | 6.53 ± 0.38 | 6.94 | 1.08 | 6.94 | 1.08 |
| 4 | CAP, MPA, 2022-A | 22 - 1 MPA | 2.15 ± 0.14 | 2.40 | 1.57 | 2.21 | 0.43 |
| 5 | CAP, MPA, 2022-A | 22 - 2 MPA | 6.59 ± 0.39 | 7.38 | 2.02 | 7.66 | 2.74 |
| 6 | CAP, MPA, 2022-A | 22 - 3 MPA | 3.19 ± 0.23 | 3.49 | 1.30 | 3.38 | 0.83 |
| 7 | CAP, MPA, 2022-B | 22 - 4 MPA | 5.38 ± 0.35 | 6.17 | 2.26 | 5.76 | 1.09 |
| 8 | CAP, MPA, 2022-B | 22 - 5 MPA | 1.47 ± 0.12 | 1.60 | 1.08 | 1.51 | 0.33 |
| 9 | CAP, MPA, 2022-B | 22 - 6 MPA | 4.17 ± 0.34 | 4.36 | 0.56 | 4.08 | 0.26 |
